# Gout, TikTok and misleading information: a content analysis

**DOI:** 10.1093/rap/rkaf126

**Published:** 2025-12-10

**Authors:** Samuela ‘Ofanoa, Siobhan Tu’akoi, Emeline Manako, Tebi Ngaire Tabokaai, Melenaite Tohi, Malakai ‘Ofanoa, Felicity Goodyear-Smith

**Affiliations:** Pacific Health Department, University of Auckland, Auckland, New Zealand; Pacific Health Department, University of Auckland, Auckland, New Zealand; Pacific Health Department, University of Auckland, Auckland, New Zealand; Division of Health, University of Waikato, Hamilton, New Zealand; Pacific Health Department, University of Auckland, Auckland, New Zealand; Pacific Health Department, University of Auckland, Auckland, New Zealand; Department of General Practice & Primary Health Care, University of Auckland, Auckland, New Zealand

**Keywords:** gout, arthritis, TikTok, social media, health education

## Abstract

**Objectives:**

This study aims to explore what types of gout content are presented on the social media platform TikTok and assess their association with user engagement.

**Methods:**

The top 200 TikTok videos captured using the search term ‘gout’ were collected. Two independent researchers coded the videos into eight main categories: account type, presenter, audio, video type, purpose, tone, overall connotation and gout content. Descriptive and inferential analyses were conducted to examine the distribution of variables and examine the association between gout content and engagement. Quotations were selected to reinforce some of the findings.

**Results:**

In total, 116 TikTok videos were included in the final analysis after excluding 84 non-relevant videos. The total number of views of the videos was ≈426.6 million, with the majority belonging to content creators from the USA. The most common presenters were patients with gout or close family members (27%). Approximately 38% of videos had negative connotations, with the most common purpose of videos being health advice (38%). The main content categories coded were management strategies (79%) and risk factors (45%), focusing overwhelmingly on diet. A significant difference in engagement was evident between gout medical sequelae and gout management (*P* < 0.05) only.

**Conclusion:**

This analysis found that there is a wide range of information being promoted on TikTok that may be misleading or inconsistent with rheumatology guidelines. Future public health strategies and health professionals have an opportunity to utilise TikTok as a platform to create content, counteract misinformation and improve public understanding of gout.

Key messages

- This study reveals that the current presentation of gout content on social media can be misleading.
- This study reinforces the limited coverage of gout urate-lowering therapy on social media platforms.
- The findings highlight the importance of health professionals and health organisations engaging social media to counteract misinformation.

## Introduction

Gout is a painful inflammatory arthritis caused by high urate in the blood that crystalises and deposits in the joints [1]. In 2017, an estimated 41 million people worldwide were affected by gout, with ≈7 million new cases diagnosed annually. This burden equates to one million years lived with disability attributable to gout [2]. Although rheumatology guidelines recommend the uptake of long-term urate-lowering therapy (ULT) for effective gout management, gout remains poorly controlled [3]. This is largely due to low adherence to optimum treatment regimens and failure to achieve target serum urate levels [3]. Previous studies have reported that there is still persisting gaps in awareness and understanding about gout among patients and the community [4, 5]. Improving education regarding what gout is and evidence-based management strategies in the community has the potential to significantly enhance gout health and quality of life [4, 6].

Social media is widely utilised as a way to connect with people and access health-related information [7, 8]. A cross-sectional survey found that 98% of participants ≥12 years of age used social media in the previous month, and those with existing health conditions (52%) were more likely to share health information [9]. A systematic review of 83 studies highlighted the increased utilisation of social media in healthcare, such as a platform for health promotion, marketing, recruitment, education, telemedicine and research [10]. This highlights the growing importance of social media as a tool for health education and engagement, particularly among younger populations.

TikTok is a social media platform that is increasingly recognised as being influential in shaping public views, perceptions and behaviours. Its format is primarily short videos that incorporate music, visual effects and other creative elements that appeal to younger audiences [11]. As of October 2023, 1.2 billion users globally engaged with TikTok, with a user base predominantly 10–29 years of age [12, 13]. A survey of 1172 women ages 18–29 years reported that ≈70% intentionally sought health information on TikTok, while 92% came across it unintentionally [14]. Although the majority of TikTok users are based in the United States (US), its popularity is rapidly expanding to other regions, including Malaysia, Saudi Arabia and the United Arab Emirates [12]. TikTok’s widespread reach and influence presents a vital opportunity for health education, including the dissemination of information about gout and its optimal management.

While previous systematic reviews and content analysis [15–19] have examined gout in academic research, health organisation communications, traditional media and various social media platforms, there remains a significant gap in the understanding of gout content specifically on TikTok. Despite TikTok’s growing influence, little is known about the nature and types of gout-related content being disseminated or how this content engages viewers. Therefore, the aim of this study is to explore what types of gout content are available on TikTok and to assess their association with user engagement. The findings for this study can help inform future strategies for public health communication and education through social media platforms.

## Methods

This study adopted a mixed-methods study design incorporating both quantitative and qualitative analysis. Given that the TikTok videos were publicly available and did not involve research on human subjects, ethics approval was not required.

### Data collection

A systematic search strategy, consistent with prior studies [20–22], was used to collect a sample of 200 TikTok videos. We searched the term ‘gout’ on the TikTok discover page and collected the first 200 videos. The sample of 200 videos was then screened and videos were excluded if they were not in the English language, if they were duplicates or if they were non-gout-related. To avoid the impacts of previous account activity and changing algorithms affecting search results, a new TikTok account was set up and all videos were collected on one day, 5 December 2024. For each included video, the following data were collected: caption, date posted, account username, number of views, likes, comments, bookmarks, shares and type of video. Creator account data comprising username, country and account type (business, health professional, health organisation, media and community) were also collected.

### Qualitative analysis

A qualitative content analysis approach was utilised to analyse the data deductively [23]. A coding framework was developed using a deductive content analysis approach similar to previous studies [16, 18, 23, 24] and validated using a preliminary analysis of the first 10 videos selected (Supplementary Table S1) [25, 26]. Consequently, the videos were categorised based on the primary type of account, presenter, audio, video type, purpose of the video, emotion/tone, overall connotation and type of gout content presented. The audio of each video was transcribed verbatim. Quotations were thematically analysed and selected to reinforce some of the findings [25]. Adopting a similar approach to Li *et al.* [27], intercoder reliability was established first by independent coding of a random sample of videos before coding the remaining sample. A random number generator was utilised to select 10% of the videos that were then independently coded by S.O., S.T., E.M. and T.T. A high Cohen’s κ value of 0.83 was achieved between reviewers and the discrepancies were then discussed to reach a consensus. The remaining videos were then coded independently by E.M. and T.T.

#### Quantitative analysis

Statistical analysis was performed using the Statistical Analysis System (SAS) version 9.4 (SAS Institute, Cary, NC, USA). Frequency and proportions were calculated for categorical variables while mean and standard deviation were computed for continuous variables. Inferential analysis was carried out to investigate differences between variables and engagement. The engagement outcome variables (views, likes, comments, shares, bookmarks) were log transformed to address skewedness due to some videos being viral. Because the log transformed variables were normally distributed, *t*-tests were used to examine associations with independent variables. The significance level was set at *P* < 0.05.

## Results

**Figure 1.**
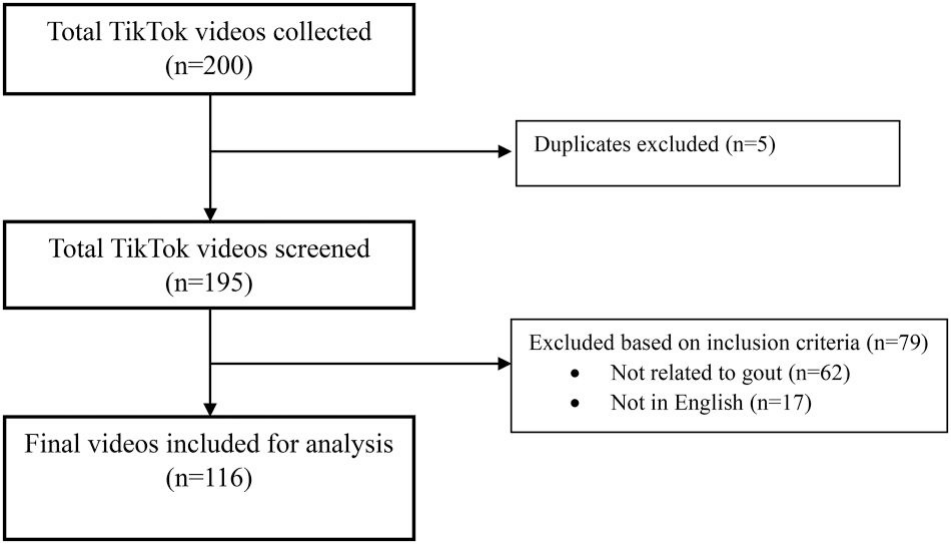
Flow diagram of the inclusion and exclusion of TikTok videos

Of 200 videos collected, 116 were deemed relevant and included in this analysis (Fig. 1). Videos garnered a total of 426.6 million views, 1.1 million likes, 343 962 comments, 102 656 shares and 90 183 bookmarks (Table 1). Content creators had a total of 59 million followers, with the majority from the USA (68%), followed by Canada, New Zealand, Australia and the Philippines—with similar proportions of 3%. The average length of videos was 52 s. Engagement in gout TikTok videos averaged 3 677 504 views (s.d. 37 056 994), 9387 likes (s.d. 59 035) and 2965 comments (s.d. 31 003), 777 bookmarks (s.d. 1900) and 885 shares (s.d. 2073).

**Table 1.** Characteristics of 116 TikTok videos.

Variables	Mean (s.d.)	Median	Total	Range
Video length (s)	52 (53)	46	6095	5–355
Log (video length)	3.57 (0.92)	3.82	414.12	1.61–5.87
Views	3 677 505 (37 056 994)	37 800	426 590 535	217–399 300 000
Log (views)	10.50 (2.32)	10.54	1218.01	5.38–19.81
Likes	9387 (59 035)	456	1 088 890	5–630 000
Log (likes)	6.23 (2.31)	6.12	722.96	1.61–13.35
Comments	2965 (31004)	19	343 962	0–334 000
Log (comments)	3.30 (2.05)	3.33	330.23	0–12.72
Bookmarks	777 (1900)	121	90 183	1–14 700
Log (bookmarks)	4.56 (2.37)	4.79	528.92	0–9.60
Shares	885 (2073)	126	102 656	0–14 900
Log (shares)	4.73 (2.39)	4.93	534.09	0–9.61
Creator account followers	508 419 (3 976 418)	8605	58 976 622	14–42 800 000
Log (creator account followers)	9.28 (2.82)	9.28	1076.61	2.64–17.57

### Video characteristics

Table 2 presents the characteristics of included videos. The main types of TikTok videos were coded as visual documentaries (36%) and oral speech (29%), while the audio type tended to be direct speech or singing (44%). Persons with gout or close family members were the most common presenters (27%), followed by health professionals (24%), members of the public (23%) and videos presented by an artificially generated voice (15%). Creator accounts were primarily owned or created by individual members of the public (53%), followed by health professionals (24%) and commercial businesses (19%)—the latter promoting the selling of gout products for profit. Consequently, the main purpose of videos was coded as providing health advice (38%), sharing personal stories (20%) and selling products (19%).

**Table 2.** Characteristics of 116 gout TikTok videos.

Characteristics	*n* (%)
Video type	
Acting or role play	11 (9.5)
Animations or illustrations	21 (18.1)
Documentary (visual)	42 (36.2)
Oral speech	33 (28.5)
Pictures or pictorial slideshows	5 (4.3)
TikTok trends or memes	4 (3.5)
Audio	
Direct spoke or singing	51 (44.0)
Voice over	26 (22.4)
Music or sounds only	25 (21.6)
No audio	2 (1.7)
More than one of the above	12 (10.3)
Presenter	
Person with gout or close family member	31 (26.7)
Health professionals	28 (24.1)
Member of the public	27 (23.3)
Artificial intelligence voice	17 (14.7)
Unclear presenter	11 (9.4)
Other	2 (1.8)
Primary type of account	
Individual member of the public	61 (52.6)
Commercial business (selling products)	22 (19.0)
Health professionals (health advice and promoting or selling supplements/herbal products)	28 (24.1)
Health organisation	2 (1.7)
Media industry	3 (2.6)
Primary purpose of video	
Entertainment	12 (10.3)
Health advice	44 (37.9)
Health education	15 (12.9)
Personal stories	23 (19.8)
Sell products (improve gout)	22 (19.0)
Overall connotation	
Positive/positive leaning	42 (35.3)
Neutral	33 (28.5)
Negative/negative leaning	41 (36.2)
Tone^a^	
Serious	43 (37.1)
Sad	4 (3.5)
Light-hearted	27 (23.3)
Hopeful	33 (28.5)
Funny	13 (11.2)
Informative	73 (62.9)

*n*: number of videos coded.

a Multiple codes were permissible.

The main tones coded in the gout videos were informative (63%) and serious (37%), and as a result, the overall connotation of the videos was slightly more negative (36%) compared with positive (35%). Negative-leaning videos often depicted pain, suffering and social embarrassment from experiences of gout flares. Humorous references were also used in many videos to joke or mock patients’ gout pain and inability to do normal daily activities. In contrast, positive-leaning videos were more hopeful, presenting certain strategies, remedies and herbal treatments that can help alleviate gout sufferers’ pain and help them cope with their symptoms.

### Gout content

Four areas of gout content were explored in this analysis: definitions of gout, risk factors, medical sequelae of gout and gout management (Table 3).

**Table 3.** Observed gout content presented in 116 TikTok videos.

Characteristics	*n* (%)
Definition of gout	*n* = 116
Yes	32 (27.59)
No	84 (72.41)
Risk factor of gout	*n* = 116
Yes	52 (44.83)
No	64 (55.17)
Types of risk factors^a^	*n* = 52
Diet and lifestyle	49 (94.2)
Medical conditions	5 (9.62)
Medications	2 (3.85)
Genetics	1 (1.92)
Ethnicity	2 (3.85)
Medical sequelae of gout	*n* = 116
Yes	9 (7.76)
No	107 (92.24)
Types of medical sequelae^a^	*n* = 9
Tophi or joint damage	5 (55.56)
Kidney impairment	4 (44.44)
Cardiovascular disease	3 (33.33)
Diabetes and insulin resistance	2 (22.22)
Hypertension	2 (22.22)
Gout management	*n* = 116
Yes	91 (78.45)
No	25 (21.55)
Types of gout management^a^	*n* = 91
Diet	48 (52.75)
Lifestyle	9 (9.89)
Supplements, herbal or home remedies	36 (39.6)
Medications	7 (7.69)
Other	6 (6.59)

a Multiple codes were permissible.

#### Definitions of gout

Approximately 28% (32/116) of TikTok videos provided a definition of gout, with the majority describing it as high urate levels in the blood that crystalise and result in gout flares. Approximately 53% (17/32) of the videos that defined gout attributed increased urate in the body to having high purine diets that included meat, sugary foods, seafood and alcohol. Only one video by a doctor stated that the majority (70%) of purines are endogenously produced within our bodies through the ‘recycling and decaying system of the cells’.

#### Risk factors and medical sequelae

Approximately 45% (52/116) of videos mentioned risk factors for gout, with diet and lifestyle being the most common (90%). The majority of these videos were presented by health professionals (52%) and individuals or family members with gout (28%). For example, a podcaster described gout as a ‘rich man’s disease, because it originated with people who eat foods with a wealthier income…you get gout from caviar and meats like steaks’. Other types of risk factors mentioned included prior medical conditions (*n* = 5), medications (*n* = 2), ethnicity (*n* = 2) and genetics (*n* = 1). One doctor used a quiz to educate the audience about risk factors that may not be commonly known, such as the effects of genetics or ethnicity and gout risk, stating, ‘Unfortunately, our people [referring to Pacific and Māori people in New Zealand] have an increased risk of getting gout, because we have genes in our body that make it difficult to get rid of uric acid’.

Medical sequelae of untreated gout were coded in only nine TikTok videos (8%). Of these, the most highlighted outcome was tophi or joint damage (*n* = 6), followed by kidney impairment (*n* = 4) and cardiovascular disease (*n* = 3). One TikTok video showed an interview with a doctor who explained, ‘if left untreated [gout], the excess uric acid will accumulate and form conditions such as tophi…this can also lead to joint problems including osteoarthritis’. Another content creator explained that ‘these high levels of uric acid are also the same thing that leads to kidney stones’. While another video linked gout to cardiovascular disease, where ‘uric acid can damage the lining of the arteries, and it can also form a lot of inflammation and oxidative stress that can damage the blood vessels and cause atherosclerosis’.

#### Gout management

Approximately 79% (91/116) of TikTok videos addressed gout management in their content, with a focus on dietary advice (*n* = 48). Some videos reported foods that could be avoided, such as one featuring a patient hospitalised for gout who advised viewers, ‘you can reduce your incidences of gout if you cut back on your salt, your alcohol and your red meat’. Other videos mentioned foods that creators believed should be consumed more; e.g. a holistic nutritionist outlined that the ‘number one is tart cherry juice. It is effective in lowering uric acid levels which, if you have gout, tend to be very high’. Supplements and herbal or home remedies were also commonly referenced types of gout management (*n* = 35), with videos promoting products such as ‘pills made from pure herbs, with no hormones and no side effects’. The majority of videos promoting herbal remedies were presented by artificial intelligent (AI)-generated voices and used hopeful emotive tones to show how viewers could beat gout naturally by drinking ‘chicory and gardenia tea…that has chicory gardenia tuckahoe dandelion and many other ingredients [that]…are very effective in helping to balance uric acid levels’. Only seven TikTok videos discussed medications as a gout management approach, primarily recommending pain relief options such as steroids and non-steroidal antiinflammatory drugs like colchicine, ibuprofen and naproxen. Just two TikTok videos mentioned ULT, with only one emphasising the importance of ‘having long-term treatment, for example allopurinol’ to avoid recurrent gout flares.

### Engagement

Table 4 presents associations between gout-related content (definition, risk factors, medical sequelae and management) and user engagement metrics (views, likes, comments, shares and bookmarks). Overall, videos that included the definition of gout, risk factors and gout management strategies showed higher mean engagement than those without. In contrast, videos discussing the consequences of gout were associated with significantly lower engagement, as shown in fewer views (mean log score of yes (M_1_) = 9.05 *vs* mean log score of no (M_2_) = 10.6, *P* = 0.01), likes (M_1_ = 4.66 *vs* M_2_ = 6.36, *P* = 0.01), comments (M_1_ = 1.67 *vs* M_2_ = 3.42, *P* = 0.01), shares (M_1_ = 3.02 *vs* M_2_ = 4.86, *P* = 0.04) and bookmarks (M_1_ = 3.11 *vs* M_2_ = 4.68, *P* = 0.01). Additionally, videos containing gout management content had significantly higher shares (M_1_ = 4.99 *vs* M_2_ = 3.69, *P* = 0.02) and bookmarks (M_1_ = 4.89 *vs* M_2_ = 3.36, *P* = 0.01) compared with those without.

**Table 4.** Engagement and gout content presented in 116 TikTok videos.

Characteristics	n	Mean (log views)	*P-*value	Mean (log likes)	*P-*value	Mean (log comments)	*P-*value	Mean (log shares)	*P-*value	Mean (log bookmarks)	*P-*value
Definition of gout											
Yes	32	10.95	0.16	6.43	0.54	3.46	0.63	5.24	0.11	4.86	0.38
No	84	10.33		6.16		3.25		4.53		4.44	
Risk factor of gout											
Yes	52	10.58	0.73	6.37	0.54	3.31	0.98	4.71	0.92	4.61	0.83
No	64	10.43		6.11		3.30		4.75		4.52	
Medical sequelae of gout											
Yes	9	**9.05**	**0.01**	**4.66**	**0.01**	**1.67**	**0.01**	**3.02**	**0.04**	**3.11**	**0.01**
No	107	**10.62**		**6.36**		**3.42**		**4.86**		**4.68**	
Gout management											
Yes	91	10.61	0.34	6.35	0.34	3.31	0.96	**4.99**	**0.02**	**4.89**	**0.01**
No	25	10.12		5.79		3.28		**3.69**		**3.36**	

Bold indicates a significant difference.

## Discussion

This content analysis aimed to explore the types of gout content available and popular on TikTok. In general, videos were primarily from US-based creators, with the main purpose of videos being to provide health education, share personal stories and sell products to improve gout health. The most coded tones of videos were informative and serious, with overall negative-leaning connotations. Presenters of the TikTok videos in this study were similar across patients (27%), health professionals (24%) and general members of the public (23%). A study by Lamb *et al.* [19] on YouTube videos and dietary recommendations found that the main source of videos were from health professionals (32%) and, as a result, the content was clinically accurate and evidence-based.

In general, TikTok videos in this analysis were found to lack accurate information about urate production and gout-related risk factors, potentially leading to misconceptions about gout. Definitions of gout varied, with only one video accurately discussing how the majority of urate in the body is produced endogenously via the breakdown and replacement of cells in the body [28, 29]. Discussions of risk factors in videos also depicted it as a disease primarily resulting from dietary factors that in turn influence urate levels. While diet and alcohol are known risk factors for gout, research emphasises that genetics, kidney impairment and weight play a significantly greater role in influencing urate levels in the body [30–32]. Content focused solely on lifestyle and dietary risk factors portrayed gout as a personal choice rather than caused by more significant underlying factors. Previous content analyses of gout content on YouTube [19] and health organisation websites [16] similarly found an emphasis on diet and lifestyle factors, often at the expense of evidence-based guidelines. Another study examining the accuracy of health information related to COVID-19 and diabetes content portrayed on 110 posts from Twitter and official Brazilian websites found that 80% were fake news, ≈12% shared incomplete results and news items [33]. This could suggest that scientific findings about gout have yet to meaningfully permeate public understanding. Incorporating more accurate information regarding urate levels and what influences them could better inform the public and shift views away from individual blame.

A disconnect was identified in this analysis whereby videos that presented gout management strategies primarily platformed advice and treatments not aligned with clinically recommended, evidence-based approaches. A total of 79% of videos in this study were coded as mentioning gout management, however, dietary advice was the most common (53%), which has limited long-term effectiveness in managing urate levels. This aligns with previous studies reviewing gout content on different media platforms, indicating that dietary interventions were often promoted, despite their lower impact [16–19]. Herbal remedies and supplements were also common gout management strategies identified in this content analysis, with videos selling products and using imagery of health professionals to increase their credibility. Bubela *et al.* [34] reported that most alternative or herbal treatments that are disseminated through newspapers often lack comprehensive reporting on clinical trial quality, contributing to public misinformation.

Notably, only two videos in this study recommended ULT for long-term gout management; however, they failed to provide important details such as on how long to take medications, side effects or medication types. This was consistent with one prior study [16] that evaluated gout patient educational resources and found that only 5% of images (14/310) depicted medications for gout. Of these, 86% of medication images were unidentifiable and only one image was of ULT—allopurinol and probenecid. Similarly, a systematic evaluation by Berch *et al.* [35] of 115 YouTube videos on gestational diabetes reported that on average most videos had a low to average (discern score = 2.7/5) score of reliability of information. A large number of videos left out key aspects of gestational diabetes management or communicated incorrect or outdated advice [35]. The underrepresentation of ULT in TikTok videos could suggest treatments based on scientific guidelines are often overshadowed by more marketable, quick-fix alternatives. A targeted health communication approach is needed, and public health strategies should prioritise the dissemination of accurate, evidence-based information on gout management through social media platforms.

A relatively small proportion of TikTok videos in this analysis (9/116) addressed the medical sequelae of untreated gout, such as tophi and joint damage, kidney impairment, cardiovascular disease, diabetes or insulin resistance and hypertension. This finding was consistent with a previous review of 115 YouTube videos on gestational diabetes, reporting low comprehensiveness (content score = 3.5/7) in the following areas: definitions, risk factors, symptoms, diagnosis, management, monitoring and complications [35]. This study also found lower engagement with these TikTok videos compared with videos that did not mention medical consequences. This may suggest users prefer other types of content about gout. For example, a study analysing the ‘Gout sufferers unite’ subreddit found that users were most commonly engaging and asking questions on diagnosis and symptom-related questions [18]. Our study found higher engagement, particularly in shares and bookmarks, for videos mentioning gout management strategies. This implies that audiences may be more inclined to engage with gout content that they perceive as more practical or relevant for managing their symptoms. The dearth of TikTok content highlighting these consequences of untreated gout may downplay the public perception of the severity of gout, potentially impacting effective gout management. This proposes the need for more targeted training to support health professionals and organisations to develop more engaging and relatable gout content.

To the best of our knowledge, this content analysis is the first to examine gout content on TikTok, contributing to the growing research base of social media and its role in public health communication. A strength of this study is the search strategy, aligned with other studies [20–22], that searched the term ‘gout’ on the TikTok discover page. This enabled the broad inclusion of videos containing gout content, beyond simply those with a hashtag, and mimicked what a typical user might see if they look for gout content on TikTok. Further, to avoid biases due to previous account activity and changing algorithms over time, a new TikTok account was created and all videos were collected over the same day. Although the study highlights gaps and opportunities for public health strategies, it is limited by the cross-sectional design, meaning that no causal inferences can be made. Additionally, only one search term (‘gout’) was used, which could result in limited videos being found. Although this research utilised strategies to avoid potential bias, TikTok’s evolving platform may create restrictions in some of the gout content reviewed in this study. The generalisability of findings may be limited by the small sample of videos in this study (*n* = 116) and the types of videos in this sample, e.g. only English-language TikTok’s were included and the majority of content creators were from the US. While focusing solely on TikTok as the social media platform for this review may introduce selection bias, as it is mainly designed for entertainment and not health education, social media can be an effective platform to influence perceptions and public understanding of health issues such as gout. As supported in the discussion section, this study was exploratory in nature and found misleading information regarding diet and management that did not align with rheumatology guidelines. Future research is needed to compare clinical recommendations and online content to explore this issue more in depth.

## Conclusion

This exploratory analysis of gout-related content on TikTok reveals a wide range of information being promoted. The majority of videos highlighted dietary risk factors and management strategies, potentially reinforcing stigma and individual blame. Herbal remedies and dietary supplements, which lack scientific validation, were also common. These findings highlight an opportunity for healthcare professionals and public health organisations to actively engage with platforms like TikTok. Innovative, engaging and evidence-based gout content may help improve our understanding of gout and its management, particularly for younger populations.

## Supplementary Material

rkaf126_Supplementary_Data

## Data Availability

Data for this study will be made available based on acceptable requests to the corresponding author.
