# Supplementary material for: Gout, TikTok and misleading information: a content analysis
Source: Rheumatol Adv Pract. 2025 Dec 10;9(4):rkaf126. doi: 10.1093/rap/rkaf126 (PMC12688442; doi:10.1093/rap/rkaf126)
Supplement: rkaf126_Supplementary_Data [file rkaf126_supplementary_data.zip › Supplementary_data gout content analysis clean.docx]

**Supplementary materials**

Supplement to: Gout, TikTok and misleading information: A content analysis

Supplementary Table S1: Coding framework utilised to analyse each TikTok video

**Supplementary Table S1: Coding framework utilised to analyse each TikTok video**

| **Code (1-3)** | **Definition** |
| --- | --- |
| **Video type** | Code only one |
| Acting or role play | Video where the information is presented by an individual or group who act or role play. |
| Animations or illustrations | Video utilises images, illustrations, graphics, cartoons or other animations to visualise gout information. |
| Documentary (visual) | Video provides a factual record or report of events or people about gout. |
| Oral speech | Video shows an individual speaking orally (often formally) about gout information to the camera. |
| Pictures or pictorial slideshows | A video showing a series of images in a pre-organised sequence. |
| TikTok trends or memes | A video showing clear Tik Tok trends and memes that other content creators follow, e.g., a gout dance or prank trend. |
| **Audio** | Code only one |
| Direct spoke or singing | Audio consists of someone directly speaking or singing to the camera. |
| Voice over | An audio voice-over with the absence of the speaker. Mainly consists of images or slideshow. |
| Music or sounds only | Video contains only music and sound audio. |
| No audio | Video containing no audio. |
| More than one audio type | Videos that contained two or more of the above categories. |
| **Presenter** | Code only one |
| Patient with gout or close family member | An individual who has gout or an immediate family member of a person with gout. |
| Health professionals | Individuals in the video who describe and present themselves as a health professional, for example general practitioners, nurses, and nutritionists |
| Member of the public | A lay member of the public who is not a health professional or person with gout. For example, community groups, or entrepreneur. |
| Artificial Intelligence (AI) | Presenter in the videos’ audio are generated or modified by or using artificial intelligence, with sounds that may portray realistic human depictions. |
| Unclear presenter | Videos that had no obvious presenter such as images or pictorial slides. |
| Public figure | A well-known figure in either entertainment or sports. |
| Media personnel | Media personnels such as podcasters, news reporters, or journalists. |
| Other | Videos that were not coded in the above categories with one or two coding such as media personnels and public figures. |
| **Primary type of account** | Code only one |
| Individual member of the public | Accounts created by lay community members |
| Commercial business | Account that is created by businesses and organisations that sell products (including gout products) for profit. |
| Health professionals | Account created by health professionals to provide health advice, and promote products. |
| Health organisation | Account that was created by a health organisation such Pharmacists or other clinical organisations. |
| Media industry | Account that was created by a media organisation that is run by journalists, news reporters or podcasters. |
| **Primary purpose of video** | Code only one |
| Entertainment | Videos with the primary purpose of use gout content to entertain the audience such as humour and music. |
| Health advice | Videos that intend to provide specific health advice about gout. |
| Health education | Videos that are intended for medical education and/or study purposes that educates and empowers people about gout. |
| Personal stories | Videos presenting on personal experiences of gout patients and/or family members such as using a certain remedy. |
| Sell products (improve gout) | Videos that intend to sell gout-related products such as supplement, herbal medications or appliances (juicers). |
| **Overall connotation** | Code only one |
| Positive/positive leaning | Videos that focused on raising awareness about gout and promoting wellness, sharing constructive tips for managing the condition. |
| Neutral | Videos that presented factual information without an emotional tone. |
| Negative/negative leaning | Videos that presented the pain and suffering of gout but also ridiculed or mocked gout. |
| **Tone** | Code all that are applicable |
| Serious | Video of gout content that presents gout messages as formal, and in a clear and concise manner. |
| Scary | Video of gout content that is viewed to evoke fear or unease. |
| Sad | Video of gout content that is perceived as causing empathy, unhappiness and sorrow. |
| Light-hearted | A video that presents gout content in cheerful, playful and easy going way. |
| Hopeful | A video that shows something positive about gout that is happening or may happen in the future. For example, not getting gout flares anymore. |
| Funny | A video that presents a humorous event or situation about gout that is intended to make people laugh. |
| Informative | Video content that aims to provide health advice and knowledge about gout. |
| **Gout content** | Code all that are applicable |
| Definition of gout | Video presents or describes the mechanisms by which gout develops such as increased uric acid in the blood and what uric acid is. |
| Risk factors | Video presents or describes characteristics or exposures that increases the likelihood of a person getting gout. |
| *Diet and lifestyle* | Food, drinks and lifestyle behaviours such as high purine meat, seafood, physical activity, drinking and smoking. |
| *Ethnicity* | Ethnicity or race is identified. |
| *Genetics* | Genetics is identified. |
| *Medical conditions* | Medical condition is identified such as diabetes, CVD, obesity, dehydration, kidney impairment. |
| *Medications* | Medications identified such as allopurinol, pain killers, |
| Medical sequalae | Video describes the association between gout and later health outcomes. |
| *Cardiovascular disease* | Cardiovascular diseases such as stroke, coronary artery disease, and rheumatic heart disease. |
| *Diabetes and insulin resistance* | Any mention of diabetes or insulin resistance. |
| *Hypertension* | Hypertension, or high blood pressure. |
| *Kidney impairment* | Any mention of kidney failure, kidney disease or kidney stones. |
| *Tophi or joint damage* | Any mention of tophi, damages to the joint, |
| Gout management | Video mentions any management strategy to manage urate levels and reduce recurrent gout flares. |
| *Diet* | Foods and drinks such as cucumbers, broccoli, cherries, water, spinach and bell peppers. |
| *Lifestyle* | Recommended lifestyle management strategies such as |
| *Medications* | Allopurinol, water pills, diuretics, |
| *Supplements, herbal or home remedies* | Any supplements, herbal or home remedies such as teas, and pills. |
| *Other* | Management strategies not otherwise discussed in above categories such as gout sleeves, acupuncture, and phlebotomy. |

**References**

1. Pleasure ZH, Becker A, Johnson DM, Broussard K, Lindberg L. How TikTok is being used to talk about abortion post-Roe: A content analysis of the most liked abortion TikToks. Contraception. 2024;133:110384.

2. Tu’akoi S, Ofanoa M, Ofanoa S, Heather M, Lutui H, Goodyear-Smith F. Exploring How Rheumatic Fever Is Portrayed on TikTok: A Descriptive Content Analysis. Int J Environ Res Public Health. 2025;22(5):686.

3. Li Y, Guan M, Hammond P, Berrey LE. Communicating COVID-19 information on TikTok: a content analysis of TikTok videos from official accounts featured in the COVID-19 information hub. Health Educ Res. 2021;36(3):261-71.
